# On Reading in Bed

**Published:** 1919-03-15

**Authors:** 


### On Reading in Bed.

When the writer was a boy reading in bed was a sin in his home, and a sin to which he was addicted. The authorities feared fire and did not approve waste of candles. Waste, forsooth! Are not joys worth any price, especially if you do not pay the price from pocket and successfully avoid payment in person? An education can be obtained between the sheets, warmly wrapped and propped by pillows, when the silence of the night has come and none will disturb you. 'Tis a time for desultory reading rather than study, but be discreet in choice of books. The exciting and enthralling is best avoided, for it tempts to undue extension of the reading hour, with loss of sleep or late hours in the morning. Arrange a table handily, and on it place a wide selection, so that you may suit your mood. More may be learnt by such browsing on books than the browser knows he learns. Do not touch on the subjects of your daily work, for time abed is time for recreation, not extension of the labour of the day; but mainly choose subjects of which you know or would know something, but from which you do not earn your bread.

